# Osteoporosis knowledge and health beliefs in ındividuals with myasthenia gravis: a cross-sectional controlled study

**DOI:** 10.1590/1806-9282.20251090

**Published:** 2026-04-20

**Authors:** Hatice Betigül Meral, Fikret Aysal, Tehran Aliyeva, Eren Toplutaş

**Affiliations:** 1 Istanbul Medipol University, Faculty of Medicine, Department of Physical Medicine and Rehabilitation – Istanbul, Turkey.; 2 Istanbul Medipol University, Faculty of Medicine, Department of Neurology – Istanbul, Turkey.

**Keywords:** Myasthenia gravis, Osteoporosis, Awareness, Knowledge

## Abstract

**OBJECTIVE:**

The aim of this study was to investigate knowledge and health beliefs regarding osteoporosis in individuals with myasthenia gravis.

**METHODS:**

This cross-sectional study included 40 individuals with myasthenia gravis and 40 healthy controls, who were selected among individuals accompanying patients at outpatient clinics and hospital support staff. Participants completed the Revised Osteoporosis Knowledge Test and the Osteoporosis Health Belief Scale. For individuals with myasthenia gravis, additional data were collected, including disease duration, current use of prednisolone, and clinical classification.

**RESULTS:**

In the myasthenia gravis group, the mean total Revised Osteoporosis Knowledge Test score was 16.6±2.3, and the mean total Osteoporosis Health Belief Scale score was 146.4±6.1, with no statistically significant difference compared with the controls (p>0.05). The myasthenia gravis group had significantly higher "barriers to exercise" scores compared with controls (p<0.001). Only 37.5% of individuals with myasthenia gravis perceived themselves to be at risk of developing osteoporosis. In the patient group, educational level showed a strong positive correlation with both total Revised Osteoporosis Knowledge Test (r=0.756, p<0.001) and total Osteoporosis Health Belief Scale (r=0.313, p=0.005) scores, whereas disease duration showed no significant correlation. There was also no significant difference in total Revised Osteoporosis Knowledge Test and Osteoporosis Health Belief Scale scores between patients currently receiving prednisolone and those not receiving it (p>0.05).

**CONCLUSION:**

This study indicates that individuals with myasthenia gravis have inadequate knowledge and low perception of osteoporosis risk. To enhance early identification and prevent osteoporosis-related complications, implementing targeted educational and preventive strategies is essential in this vulnerable population.

## INTRODUCTION

Osteoporosis is a skeletal disorder marked by low bone mass and structural deterioration, increasing fragility and fracture risk^1^. Its asymptomatic course and link to morbidity and mortality make it a major health concern, especially in the elderly. In addition to age and menopause, osteoporosis frequently develops secondary to chronic illnesses and prolonged use of certain medications, including glucocorticoids, anticonvulsants, selective serotonin reuptake inhibitors, and proton pump inhibitors^2^. Among secondary causes, long-term glucocorticoid use is a key risk factor^3^. While effective in autoimmune disease management, glucocorticoids impair bone metabolism, leading to bone mineral density (BMD) loss and fractures^4^. Glucocorticoid-induced osteoporosis (GIO), reported even at low doses, is associated with disability and poor quality of life^5,6^.

Myasthenia gravis (MG) is the most common autoimmune neuromuscular disorder, characterized by fluctuating muscle weakness and fatigue, primarily caused by autoantibodies against the acetylcholine receptor (AChR) or muscle-specific kinase (MuSK)^7^. The incidence of MG has been rising globally, particularly among older adults, increasing the clinical complexity and comorbidity burden in this population. Glucocorticoids are frequently used in the long-term management of MG to suppress autoantibody-mediated immune responses.

In the literature, the number of studies evaluating the relationship between glucocorticoid use and osteoporosis in individuals with MG has been increasing^8,9^. In this patient group, high-dose and long-term oral glucocorticoid therapy has been reported to increase the risk of major osteoporotic fractures, and some studies have suggested that this risk may be more strongly associated with the duration of use rather than the cumulative dose^10,11^. Moreover, although MG does not directly compromise bone health, it can lead to secondary risk factors such as reduced mobility, further compounding the risk of osteoporosis and fractures in this population^12^.

Given that GIO can be largely prevented through lifestyle modifications and pharmacological interventions, evaluating patients’ knowledge regarding osteoporosis is crucial for developing effective preventive strategies and reducing long-term complications such as fractures and disability. This study aims to assess both knowledge and health beliefs regarding osteoporosis in individuals with MG. To our knowledge, this is the first study in the literature on this subject in the MG population.

## METHODS

### Study design and participants

This cross-sectional controlled study was conducted between May 1 and June 1, 2025, and included individuals diagnosed with MG as well as age- and sex-matched healthy controls. Participants with MG were recruited from neurology and physical medicine and rehabilitation outpatient clinics.

The diagnosis of MG was based on clinical presentation supported by at least one of the following: a positive response to intravenous acetylcholinesterase inhibitors, a decremental response on low-frequency repetitive nerve stimulation, or the presence of autoantibodies against AChR or MuSK^13^. Inclusion criteria for the MG group were age ≥18 years, confirmed diagnosis of MG, and sufficient cognitive capacity to complete the assessments. Exclusion criteria included a prior diagnosis of osteoporosis, other chronic bone diseases, known cognitive impairments or dementia affecting accurate responses, and being pregnant or breastfeeding, as these physiological conditions can significantly affect bone metabolism and confound osteoporosis-related assessments.

Healthy controls were selected among individuals accompanying patients at the outpatient clinics and among hospital support staff (such as cleaning, security, food service, and technical support personnel). All control participants were screened via self-report and medical records to rule out neuromuscular, metabolic, or chronic bone diseases, as well as long-term medication use that could affect bone metabolism. In addition, participants with a history of glucocorticoid use, pregnancy, or lactation were excluded. To ensure comparability between groups, matching was performed based on age (±3 years) and sex.

As a result, the study sample comprised 40 individuals with MG and 40 healthy controls.

### Data collection

Sociodemographic and clinical characteristics of the participants were recorded, including age, sex, menopausal status (in female participants), education level, and body mass index (BMI). Education level was categorized as primary school, middle school, high school, and university or above.

For individuals with MG, disease-specific variables were also recorded, including disease duration and current use of prednisolone. Patients were also classified according to the Myasthenia Gravis Foundation of America (MGFA) Clinical Classification, which categorizes patients based on the distribution and severity of muscle weakness^14^. Moreover, two specific questions were included in the data collection process to evaluate osteoporosis awareness: "Do you know what osteoporosis is?" and "Do you think you are at risk of osteoporosis?" The participants responded to these questions with "Yes," "No," or "I don't know."

### Assessment tools

We administered three validated instruments to evaluate participants’ knowledge and health beliefs regarding osteoporosis.

The Revised Osteoporosis Knowledge Test (R-OKT) is a 32-item instrument consisting of two subscales: nutrition (26 items) and exercise (20 items), with 14 items overlapping between the two domains. The score range for the nutrition subscale is 0–26, while that for the exercise subscale is 0–20. Participants are asked to indicate whether each statement is related to the development of osteoporosis. All questions are presented in multiple-choice format. Correct answers are scored as 1, while incorrect or "don't know" responses are scored as 0. The total score ranges from 0 to 32, with higher scores reflecting greater knowledge about osteoporosis. A classification scheme introduced by Vered et al. suggests that R-OKT scores above 25.6 reflect high knowledge, scores between 19.2 and 25.6 reflect moderate knowledge, and scores below 19.2 indicate insufficient knowledge^15,16^. The Turkish version was validated by Atalay et al^17^.

The Osteoporosis Health Belief Scale (OHBS) is a 42-item self-report tool assessing beliefs across seven subdomains, including susceptibility, seriousness, benefits, barriers, and motivation. The susceptibility subscale aims to assess individuals’ perceived risk of developing osteoporosis. It includes items that evaluate perceptions related to two major risk factors: body build and family history, as well as the perceived likelihood of developing osteoporosis. The seriousness subscale measures the extent to which individuals perceive osteoporosis as a threat to their ability to carry out daily activities, maintain physical health, and preserve their social status. Items related to exercise assess beliefs about the role of physical activity in preventing osteoporosis, the effects of regular exercise on bone health, and the individual's attitudes toward exercise in this context. Items related to calcium intake evaluate the belief that sufficient calcium consumption reduces the risk of developing osteoporosis and associated fractures.

Items are rated on a six-point Likert scale, with higher scores indicating stronger health beliefs. Subscale scores range from 6 to 30, total scores from 42 to 210. Based on each domain score, participants’ health beliefs were classified into five categories: low (6–11), moderately low (12–16), neutral (17–19), moderately high (20–24), and high (25–30)^18^. The Turkish version has been validated^19^.

This research was approved by the institution's Research Ethics Committee (Decision No: E-10840098-202.3.02-2622), on 21/04/2025.

### Statistical analysis

The data were analyzed using IBM SPSS Statistics for Windows, version 23.0 (IBM Corp., Armonk, NY, USA). Continuous variables were summarized as mean±standard deviation or median, and categorical variables as frequency and percentage. Normality was assessed via the Shapiro-Wilk test. Since most variables were non-normally distributed, the Mann-Whitney U test and chi-square test were used to compare groups. Spearman correlation analysis was performed. Effect sizes were calculated for each test: rank-biserial correlation for the Mann-Whitney U test and Cramer's V for the chi-square test. Correlation strength was interpreted as weak (0.10–0.39), moderate (0.40–0.69), strong (0.70–0.89), and very strong (0.90–1.00)^20^. A p<0.05 was considered statistically significant.

An a priori power analysis was conducted. With the current sample (n_1_=40, n_2_=40), the sensitivity analysis indicated that the minimum detectable effect size at 80% power is d=0.64. This value suggests that the study is adequately powered to detect medium-to-large effects.

## RESULTS

A total of 40 individuals with MG and 40 age- and sex-matched healthy controls were included in the study. The demographic and clinical characteristics of the participants are presented in Table 1. There were no statistically significant differences between the groups in terms of age, sex, menopausal status, BMI, or educational level. Among individuals with MG, 65% were currently using prednisolone.

**Table 1:** Demographic and clinical characteristics of myasthenia gravis patients and controls.

Variables		MG Patients		Controls		p	Effect size
		n (%)	Mean±SD	n (%)	Mean±SD		
Age (year)			50.8±7.5		50±6.9	0.620*	0.065^a^
Sex							
	Female	25 (62.5)		24 (60)		0.818**	0.026^b^
	Male	15 (37.5)		16 (40)			
	Postmenopause	12 (30)		14 (35)		0.469**	0.104^b^
	BMI (kg/m²)		24.5±2		24.2±1.7	0.372*	0.081^a^
Education level							
	Primary school	8 (20)		5 (12.5)		0.612**	0.185^b^
	Middle school	6 (15)		10 (25)			
	High school	13 (32.5)		9 (22.5)			
	University or above	13 (32.5)		16 (40)			
	Current use of PSL	26 (65)					
MGFA classification							
	Class I	9 (22.5)					
	Class II	20 (50)					
	Class III	11 (27.5)					
Do you know what OP is?							
	Yes	22 (55)		19 (47.5)		0.605**	0.112^b^
	No	9 (22.5)		8 (20)			
	I don't know	9 (22.5)		13 (32.5)			
Do you think you are at risk of OP?							
	Yes	15 (37.5)		10 (25)		0.438**	0.144^b^
	No	11 (27.5)		15 (37.5)			
	I don't know	14 (35)		15 (37.5)			

BMI: body mass index; PSL: prednisolone; MGFA: Myasthenia Gravis Foundation of America; OP: osteoporosis; MG: Myasthenia gravis; SD: standard deviation.

* Mann-Whitney U test,

** Chi-square test.

a Rank-biserial correlation,

b Cramer's V.

The proportion of participants who answered "Yes" to the question "Do you know what osteoporosis is?" was 55% in the MG group and 47.5% in the control group (p=0.605). Similarly, 37.5% of individuals with MG and 25% of controls reported believing they were at risk for osteoporosis (p=0.438).

There were no significant differences between the groups in the total or subdomain scores of the R-OKT, including exercise and nutrition scores. Likewise, most subdomains of the OHBS did not differ significantly between groups. However, the OHBS "Barriers to Exercise" score was significantly higher in individuals with MG (15.8±1.7) compared to controls (14.3±1.6) (p<0.001) (Table 2).

**Table 2:** Comparison of Osteoporosis Knowledge Test and Osteoporosis Health Belief Scale scores between myasthenia gravis patients and the control group.

Scales		MG patients			Control group				Effect size^a^
		Mean±SD	Median	IQR	Mean±SD	Median	IQR	p*	
OKT									
	Exercise	8.9±1.7	9	2	8.8±1.4	9	2	0.691	0.051
	Nutrition	12.7±2	13	2	12.6±1.2	13	1	0.258	0.143
	Total	16.6±2.3	17	2.25	16.3±1.4	16	2	0.412	0.106
OHBS									
	Susceptibility	20.4±2.2	20	3	20±2.1	20	2	0.467	0.058
	Seriousness	21.9±2	22	2	22.1±1.7	22	2	0.672	0.009
	Benefits of exercise	25.9±1.3	26	2	26.3±1.7	26	2	0.234	0.151
	Benefits of calcium intake	24.5±1.7	24	2	24.9±2	24.5	1	0.473	0.091
	Barriers to exercise	15.8±1.7	16	2	14.3±1.6	14	2	**<0.001**	0.468
	Barriers to calcium intake	13.1±1.6	13	2	12.8±2.2	12.5	2	0.115	0.202
	Health motivation	24±1.3	24	3	24±1.5	24	3	0.952	0.008
	Total	146.4±6.1	146	4.25	144.7±5.7	146	5.50	0.484	0.091

Note: Values of p<0.05 were accepted as significant and are marked in bold. MG: myasthenia gravis; OKT: Osteoporosis Knowledge Test; OHBS: Osteoporosis Health Belief Scale; SD: standard deviation; IQR: interquartile range.

* Mann-Whitney U test,

a Rank-biserial correlation.

Within the MG group, disease duration was not significantly correlated with either the total R-OKT score (r=0.094, p=0.562) or the total OHBS score (r=0.292, p=0.067). In contrast, educational level showed a strong positive correlation with both total R-OKT (r=0.756, p<0.001) and OHBS scores (r=0.313, p=0.005) (Table 3).

**Table 3:** Correlation between myasthenia gravis patients’ disease duration, educational status, and scale scores.

Scales		Disease duration		Education level	
		R	P	r	p*
Osteoporosis Knowledge Test					
	Exercise	0.014	0.930	0.775	**<0.001**
	Nutrition	0.065	0.688	0.681	**<0.001**
	Total	0.094	0.562	0.756	**<0.001**
Osteoporosis Health Belief Scale					
	Susceptibility	0.391	**0.013**	0.347	**0.002**
	Seriousness	0.470	**0.002**	0.234	**0.037**
	Benefits of exercise	-0.442	**0.004**	0.219	0.052
	Benefits of calcium intake	-0.085	0.602	0.153	0.176
	Barriers to exercise	0.185	0.254	-0.029	0.798
	Barriers to calcium intake	0.629	**<0.001**	0.119	0.292
	Health motivation	0.062	0.703	0.005	0.964
	Total	0.292	0.067	0.313	**0.005**

Note: Values of p<0.05 were accepted as significant and are marked in bold.

* Spearman correlation test.

Within the MG group, comparison of patients currently using prednisolone (n=26) and those not using it (n=14) revealed no significant differences in osteoporosis knowledge. Total R-OKT scores and its subdomains did not differ significantly between the two groups (p>0.05) (Table 4). Similarly, no statistically significant difference was observed in the total OHBS scores between prednisolone users and non-users (p=0.055) (Table 4). However, four OHBS subdomains showed statistically significant differences. Prednisolone users reported higher perceived susceptibility to osteoporosis (p=0.016), as well as greater perceived barriers to exercise (p=0.016) and barriers to calcium intake (p=0.006). In addition, their scores for perceived benefits of exercise were significantly lower compared to non-users (p=0.006).

**Table 4:** Comparison of Osteoporosis Knowledge Test and Osteoporosis Health Belief Scale scores between Myasthenia gravis individuals with and without current prednisolone use.

Glucocorticoid exposure									
Scales		Current prednisolone use (+)			Current prednisolone use (-)			p*	Effect size^a^
		(n=26)			(n=14)				
		Mean±SD	Median	IQR	Mean±SD	Median	IQR		
OKT									
	Exercise	8.65±1.77	9.00	1.00	9.43±1.60	10.00	0.00	0.113	0.346
	Nutrition	12.62±1.96	13.00	1.00	12.93±1.98	14.00	2.50	0.379	0.195
	Total	16.38±2.42	16.00	1.00	16.86±1.99	18.00	2.50	0.402	0.173
OHBS									
	Susceptibility	21.04±2.24	21.00	2.00	19.14±1.51	19.50	1.75	**0.016**	0.541
	Seriousness	22.31±2.02	22.50	1.00	21.21±1.72	21.00	1.00	0.066	0.401
	Benefits of exercise	25.46±1.21	25.00	1.00	26.79±0.98	27.00	0.00	**0.006**	0.654
	Benefits of calcium intake	24.35±1.72	24.00	2.00	24.86±1.51	25.00	1.50	0.235	0.261
	Barriers to exercise	16.31±1.52	16.50	1.75	14.71±1.68	14.50	1.00	**0.016**	0.525
	Barriers to calcium intake	13.88±1.31	14.00	1.75	11.71±0.91	12.00	0.75	**0.006**	0.879
	Health motivation	23.88±1.18	24.00	1.50	24.29±1.38	24.50	2.00	0.403	0.159
	Total	147.96±6.06	146.50	4.00	143.57±5.33	144.50	2.00	0.055	0.434

Note: Values of p<0.05 were accepted as significant and are marked in bold. OKT: Osteoporosis Knowledge Test; OHBS: Osteoporosis Health Belief Scale; SD: standard deviation; IQR: interquartile range.

* Mann-Whitney U test,

a Rank-biserial correlation.

## DISCUSSION

The results of this study indicated that both individuals with MG and healthy controls demonstrated insufficient knowledge about osteoporosis. A low risk perception regarding osteoporosis was observed among individuals with MG. Among individuals with MG, educational status showed a positive correlation with both osteoporosis-related knowledge and health beliefs. Total knowledge and health belief scores did not differ significantly between patients currently using prednisolone and those not using it. However, those currently on prednisolone reported lower perceived benefits of exercise and faced greater barriers to both exercise and calcium intake.

In the treatment of MG, long-term glucocorticoid therapy is frequently used and remains effective for symptom control; however, it increases the risk of reduced BMD and fragility fractures. This is clinically relevant, as GIO is among the most common forms of secondary osteoporosis observed in chronic autoimmune disorders. Konno et al. emphasized that GIO has a significant negative impact on health-related quality of life in individuals with MG, underscoring the clinical importance of skeletal monitoring in this population^11^. Supporting these findings, a recent study reported that 17.9% of individuals with MG on long-term corticosteroid therapy experienced osteoporotic fractures over a 10-year period, further highlighting the real-world impact of GIO^21^. In this context, a noteworthy finding of our study was that individuals with MG demonstrated inadequate levels of osteoporosis knowledge and a low perception of related risks. This may be associated with insufficient awareness regarding the potential skeletal effects of glucocorticoid therapy and the limited emphasis on patient education in current clinical practice. Therefore, integrating structured osteoporosis education programs into MG management protocols is of great importance. Furthermore, since GIO often develops insidiously and remains asymptomatic until fractures occur, regular BMD screening should be considered a fundamental component of disease management in this population.

In our study, patients’ beliefs regarding osteoporosis were assessed using the OHBS, which is based on the Health Belief Model^22^. One of the key findings of the study was that the perceived barriers to exercise—reflecting individual difficulties and attitudes toward physical activity independent of osteoporosis knowledge—were significantly higher in the MG group compared to the control group. With regard to the subgroup analysis, individuals currently using prednisolone were found to have significantly lower beliefs in the benefits of exercise and higher perceived barriers to exercise compared to non-users. Although several studies in the literature have reported that individuals with MG have lower levels of physical activity compared to the general population, research evaluating their attitudes and perceptions toward physical activity remains quite limited^23,24^. In a study conducted in Australia, Alsop et al. explored the perspectives of individuals with MG regarding physical activity and their experiences with professional guidance^25^. Participants generally expressed a positive attitude toward physical activity; however, they also reported difficulties in maintaining regular activity due to fatigue, pain, and limited support. Given that patients undergoing active glucocorticoid therapy are more likely to experience symptoms such as fatigue and muscle weakness more intensely, the more distant and unmotivated attitudes toward physical activity observed in these patients in our study may be associated with the physical and psychological effects of long-term glucocorticoid use.

In the same study, Alsop et al. also noted that the majority of participants did not receive adequate, disease-specific exercise recommendations from healthcare professionals and were forced to develop their own coping strategies to remain active^25^. One of the underlying reasons for this situation may be that both patients and healthcare professionals have historically approached physical activity with caution due to symptoms commonly observed in MG, such as muscle weakness, fatigue, and low exercise tolerance^26^. However, recent studies have shown that exercise is safe for patients with clinically stable MG, and that regular physical activity may exert beneficial effects on both physical and psychosocial health outcomes^27,28^.

From the perspective of osteoporosis, physical activity is a fundamental factor in maintaining bone health and reducing fracture risk. Structured exercise programs incorporating walking, resistance, balance, posture, and flexibility training help prevent falls and increase bone mineral density, thereby reducing osteoporotic fractures^29^. Considering all these findings, it becomes evident that healthcare professionals must not only act as guides but also take on an active, supportive, and implementing role in the development and dissemination of patient-centered, applicable, and sustainable exercise programs that account for the unique physical limitations, psychosocial barriers, and individual differences of individuals with MG.

A positive association between educational level and osteoporosis-related knowledge has been demonstrated in numerous studies^30,31^. Consistent with the literature, our study also revealed a strong positive correlation between patients’ educational level and their knowledge about osteoporosis. This finding suggests that an individual's educational background may be an important determinant not only of knowledge level but also of health literacy, access to information, and coping abilities. Therefore, educational level should be considered during clinical assessment, and risk stratification and intervention strategies should be tailored accordingly to optimize patient management. At this point, visually supported and interactive approaches based on materials developed by the International Osteoporosis Foundation and national endocrinology and neuromuscular associations may assist clinicians in making patient information processes more accessible and effective.

This study has several limitations. The most significant limitation is the absence of detailed data on cumulative glucocorticoid exposure. Only current use was recorded, with no information on dose, treatment duration, or prior use. Because of this limitation, glucocorticoid exposure—the primary clinical risk factor for osteoporosis in MG—could not be accurately quantified, and the relationship between glucocorticoid therapy and osteoporosis knowledge could not be fully interpreted. Future studies should collect comprehensive and standardized data on glucocorticoid dose and duration to enable a more precise evaluation of these associations. In addition, the cross-sectional design of the study restricts causal inference. Considering that the statistical power was limited due to the sample size and was underpowered to detect small-to-medium effect sizes, the potential risk of a Type II error should be taken into account. Comorbidities and prior osteoporosis education were also not fully controlled.

In our study, the control group consisted of hospital support staff and patient companions who were generally not medically trained. However, considering that these individuals may have greater baseline health literacy and exposure to medical information compared to the general population, this selection could introduce a potential bias. This may have led to an underestimation of the true knowledge gap between individuals with MG and the general public. Future studies should aim to minimize this potential bias by recruiting control participants from a more representative sample of the general population.

In conclusion, the findings of this study demonstrate a lack of sufficient knowledge and risk perception regarding osteoporosis among individuals with MG, who are vulnerable to bone health complications. These findings highlight the need for tailored educational interventions, sustained awareness efforts, and proactive preventive strategies specifically targeting this high-risk population. Future research should adopt longitudinal designs and comprehensively evaluate both clinical and behavioral determinants to better understand and address this critical gap in osteoporosis care.

## Data Availability

The datasets generated and/or analyzed during the current study are available from the corresponding author upon reasonable request.
